# Oxidative damages in tubular epithelial cells in IgA nephropathy: role of crosstalk between angiotensin II and aldosterone

**DOI:** 10.1186/1479-5876-9-169

**Published:** 2011-10-06

**Authors:** Joseph CK Leung, Loretta YY Chan, Sydney CW Tang, Man-Fai Lam, Chui-Wa Chow, Ai-Ing Lim, Kar-Neng Lai

**Affiliations:** 1 Department of Medicine, Queen Mary Hospital, University of Hong Kong, Pokfulam, Hong Kong

## Abstract

**Background:**

Inhibition of the renin-angiotensin-aldosterone system (RAAS) slows down the progression of chronic renal diseases (CKD) including IgA nephropathy (IgAN). Herein, we studied the pathogenetic roles of aldosterone (Aldo) in IgAN.

**Methods:**

Human mesangial cells (HMC) was activated with polymeric IgA (pIgA) from IgAN patients and the effects on the expression of RAAS components and TGF-β synthesis examined. To study the roles of RAAS in the glomerulotubular communication, proximal tubular epithelial cells (PTEC) was cultured with conditioned medium from pIgA-activated HMC with eplerenone or PD123319, the associated apoptotic event was measured by the generation of nicotinamide adenine dinucleotide phosphate (NADPH) oxidase and reactive oxygen species (ROS).

**Results:**

Polymeric IgA up-regulated the Aldo synthesis and aldosterone synthase expression by HMC. The release of TGF-β by HMC was up-regulated synergistically by AngII and Aldo and this was inhibited by incubation of HMC with losartan plus eplerenone. Cultured PTEC express the mineralocorticoid receptor, but not synthesizing aldosterone. Apoptosis, demonstrated by cleaved PARP expression and caspase 3 activity, was induced in PTEC activated by conditioned medium prepared from HMC cultured with pIgA from IgAN patients. This apoptotic event was associated with increased generation of NADPH oxidase and ROS. Pre-incubation of PTEC with PD123319 and eplerenone achieved complete inhibition of PTEC apoptosis.

**Conclusions:**

Our data suggest that AngII and Aldo, released by pIgA activated HMC, served as mediators for inducing apoptosis of PTEC in glomerulo-tubular communications. Crosstalk between AngII and Aldo could participate in determining the tubular pathology of IgAN.

## Background

IgA nephropathy, the most common primary glomerulonephritis worldwide, is associated with a substantial risk of progression to end-stage renal failure (ESRF) [1]. The disease runs a highly variable clinical course. A subgroup of IgAN with tubulointerstitial damage is often associated with the most rapid progression to ESRF [2]. We have previously documented that mesangial IgA deposition induces local release of pro-inflammatory cytokines leading to glomerular inflammation [3,4]. The renin-angiotensin system (RAS) is strongly involved in the development of progressive renal fibrosis with local AngII hyperactivity occurring in IgAN [5-7]. We had revealed that IgA from IgAN patients was capable of up-regulating the TGF-β production *via *increased AngII release by HMC following binding to pIgA [8]. We further demonstrated altered expression of mesangial and tubular angiotensin receptors in response to raised intra-renal AngII in IgAN [3,4,9]. Although these data shed light on the importance of AngII and RAS in the pathogenesis of IgAN, a possible link between the aldosterone system and IgAN remains lacking. Aldosterone is an important mediator bearing injurious actions of the RAAS in chronic heart failure and renal disease [10-13]. Aldo promotes fibrosis and vascular toxicity in experimental animal models [14-16]. The specific action of Aldo is mediated through the mineralocorticoid receptor (MR) in the presence of 11ß-hydroxysteroid dehydrogenase type II (11ß-HSD2) [13]. In humans, exogenous aldosterone increases circulating interleukin-6 (IL-6) concentrations and MR antagonism attenuates AngII-induced IL-6 increase [17], suggesting that endogenous aldosterone may contribute to the pro-inflammatory effects of AngII. AngII inhibition combined with Aldo blockade effectively reduces proteinuria in human CKD [18]. All these evidences suggest that Aldo may also be involved in the pathophysiology of IgAN.

## Methods

### Materials

Reagents used for cell culture were obtained from Life Technologies (Rockville, MD, USA). Monoclonal anti-MR was obtained from Abcam (Cambridge, MA, USA). Rabbit anti-11ß-HSD2 was obtained from Cayman Chemical (Ann Arbor, MI, USA). Goat anti-CYP11B2, rabbit polyclonal anti-AT1R (AT1R) and AngII receptor subtype-II (AT2R) were from Santa Cruz Biotechnology (Santa Cruz, CA, USA). Monoclonal anti-cleaved poly-(ADP-ribose)-polymerase (PARP) was obtained from Cell Signaling Technology (Beverly, CA, USA). Monoclonal anti-actin was obtained from Neomarkers (Fremont, CA, USA). F(ab')_2 _fragment of Alexa Fluor 488-conjugated goat anti-mouse, donkey anti-goat, goat anti-rabbit immunoglobulin G (IgG) antibodies were obtained from Invitrogen Ltd. (Paisley, UK). The Envision Plus System was obtained from Dako (Carpinteria, CA, USA). Peroxidase labeled anti-goat antibody was obtained from Jackson ImmunoResearch Laboratories, Inc. (West Grove, PA, USA). 4',6'-diamidino-2-phenylindole hydrochloride (DAPI) was obtained from Molecular Probe (Eugene, OR, USA). Human total kidney RNA was obtained from Life Technologies Corporation (Carlsbad, CA, USA). Angiotensin II, aldosterone, angiotensin-converting enzyme inhibitor (ACEI), eplerenone, AngII receptor antagonist and other chemicals were obtained from Sigma (St. Louis, MO, USA).

### Study Population

The study protocol was approved by the Research Ethics Committee of the University of Hong Kong and was carried out in accordance with the principles of Declaration of Helsinki. Twenty-seven Chinese patients (12 male and 15 female) with clinical and renal immunopathological diagnosis of primary IgAN were studied. Fifty milliliters of blood were collected from each studied subject at clinical quiescence (no macroscopic hematuria with urinary erythrocyte count < 10,000/ml in un-centrifuged urine). The serum was isolated and frozen at -20°C until for isolation of pIgA1. Twenty-two healthy subjects (10 male and 12 female), comparable in age and race, with no microscopic hematuria or proteinuria, were recruited as controls.

### Purification of pIgA

Jacalin binding protein (JBP) was purified using a jacalin-agarose affinity column and pIgA was fractionated by the FPLC as described previously [19].

### Cell Culture and Preparation of Conditioned Medium

Isolation and characterization of HMC and PTEC were performed as previously described [4,20]. Growth arrested HMC were cultured in six-well culture plates (1 × 10^6 ^cells per well) with culture medium containing 0.5% FBS and pIgA (final concentration 0.5 mg/ml) isolated from patients with IgAN or controls for 48 hours. The conditioned medium (IgA-HMC medium) after culture were collected and kept frozen at -70°C until used. Conditioned medium from HMC cultured without the addition of IgA preparation was used as plain medium control.

### Expression of MR, 11β-HSD2 or CYP11B2 in HMC and PTEC

HMC or PTEC were cultured with pIgA (0.5 mg/ml) from controls or IgAN patients, or IgA-HMC medium from IgAN patients (for PTEC only), for 6 h (for RNA expression) or 24 h (for protein determination). The expression of MR, 11β-HSD2 and CYP11B2 were determined by qPCR and immunoblotting. Expression of CYP11B2 in HMC or MR in PTEC was also examined using immunofluorescence staining.

### Cell Culture Experiments

To determine the dose- or time-course of CYP11B2 expression or Aldo synthesis by HMC, growth arrested HMC were cultured with pIgA (0.125 to 2 mg/ml) or AngII (10^-12 ^to 10^-8 ^M) for 6 h (for RNA expression) or 48 h (for protein or Aldo assay); or with pIgA (0.5 mg/ml) or AngII (10^-10 ^M) for 6 h to 96 h. To determine the dose course of angiotensinogen and ACE expression or AngII release by HMC, growth arrested HMC were cultured with Aldo (10^-12 ^to 10^-8 ^M) for 6 h (total RNA purification) or 48 h (for AngII assay). To examine whether there was synergistic effect of AngII and Aldo on TGF-β synthesis by HMC, growth arrested HMC were cultured with different concentrations of Aldo, AngII or their combination (10^-12 ^to 10^-8 ^M) for 48 h. For studying the role of MR and AT1R in pIgA induced TGF-β synthesis by HMC, HMC were pre-incubated with losartan (100 mM) or/and eplerenone (10 μM) one hour before addition of pIgA.

To examine the differential effect of pIgA, conditioned medium, AngII and Aldo on expression of MR or AT2R, ROS generation and NADPH oxidase activity by PTEC, growth arrested PTEC were cultured with (i) pIgA (0.5 mg/ml) from IgAN patients; (ii) 4 fold diluted IgA-HMC medium prepared from controls or IgAN patients; (iii) Aldo (10^-10 ^M) or (iv) AngII (10^-10 ^M) for 6 h (for mRNA) or 48 h (for protein). PTEC were also pre-incubated with losartan (100 mM), PD123319 (10 μM), eplerenone (10 μM) or their combinations one hour before experiments for studying the role contributed by MR, AT1R or AT2R in ROS generation and caspase 3 activity induced by conditioned medium or AngII.

To determine the time-course of cleaved PARP or caspase 3 activity in PTEC, PTEC were cultured with (i) pIgA (0.5 mg/ml) from IgAN patients; (ii) 4 fold diluted IgA-HMC medium prepared from controls or IgAN patients; (iii) Aldo (10^-10 ^M) or (iv) AngII (10^-10 ^M) for different time points For dose-course study, PTEC were cultured with various concentrations or dilutions of (i) pIgA from IgAN patients; (ii) IgA-HMC from controls or IgAN patients; (iii) Aldo or (iv) AngII for 48 h.

### Immunohistochemistry Examination

Renal tissues were obtained from eight normotensive patients with mild IgAN (grade 1) consecutively admitted for diagnostic renal biopsy with the presentation of microscopic haematuria. They had not previously received angiotensin-converting enzyme inhibitor or Ang II receptor subtype-1 (ATR1) antagonist. Control renal tissues were obtained from the intact pole of kidneys removed for single circumscribed tumor in seven normotensive subjects (comparable in age, sex and race). Glomerular and tubular expression of MR, 11β-HSD2 and CYP11B2 were detected by immunoperoxidase staining using specific antibodies. The bound monoclonal anti-MR or rabbit polyclonal anti-11β-HSD2 antibodies were visualized in brown using the Dako Envision Plus System. The bound goat anti-CYP11B2 antibodies were detected with peroxidase labeled anti-goat antibodies and visualized in brown using 3,3'-diaminodbenzidine (DAB). Negative controls were done with primary antibodies absorbed with relevant antigens before the first incubation. Two renal pathologists without prior knowledge of clinical or laboratory data evaluated the expression of MR, 11β-HSD2 and CYP11B2 using an arbitrary 0-5+ scale [4,9].

### Immunoblotting

HMC or PTEC were harvested and dissolved in protein extraction buffer containing protease inhibitor cocktails. Total protein from HMC (50 μg) or PTEC (10 μg) was electrophoresed through a 15% SDS-PAGE and then transferred to the polyvinylidene fluoride (PVDF) membrane. The membrane was incubated overnight with anti-CYP11B2 (1:1000), anti-AT2R (1:1000), anti-MR (1:1000) or anti-actin (1:1000) antibody in PBS-Tween before reacting with appropriate peroxidase-labeled secondary antibodies (Dako, Kyoto, Japan). The reaction was detected with ECL plus chemiluminescent detection reagent (Amersham Pharmacia Biotech, Uppsala, Sweden). The images were scanned and the density of the bands was quantitated using the ImageQuant software (Molecular Dynamic, Sunnyvale, CA, USA). The densitometric results were reported as average arbitrary integrated values (units) after normalization with the average arbitrary integrated values of the actin signal.

### Detection of Cleaved PARP Expression in HMC and PTEC

Cell extracts were prepared from HMC or PTEC. Early apoptosis was detected by immunoblotting using monoclonal anti-PARP antibody (1:500) that recognized the 89 kDa cleaved PARP fragment. The membrane was then washed and incubated for 2 hours at room temperature with a peroxidase-labeled goat anti-mouse immunoglobulin (Dako, Kyoto, Japan). The reaction was quantitated as described in the previous section.

### Real-time RT-PCR

Real-time RT-PCR was performed as previously described [21]. Primer sequences and gene bank accession numbers are listed in Table 1. Data obtained were analyzed using the comparative *C*_T _(cycle threshold) method.

**Table 1:** Primer sequences for qPCR

Gene	Forward primer sequences	Reward primer sequences
CYP11B2	5'-TACAGGTTTTCCTCTACTCG	5'-AGATGCAAGACTAGTTAATC
11β-HSD2	5'-GGCCACAATGAAGTAGTTGC	5'-CTCCCCACAGTCACGATG
ANG	5'-CTGCAAGGATCTTATGACCTGC	5'-TACACAGCAAACAGGAATGGGC
ACE	5'-CCGAAATACGTGGAACTCATCAA	5'-CACGCGTCCCCTGCATCTACA
MR	5'-TCTGACTCTGGGAGCTCCGT	5'-TCCTCCTAGACATGAGCTGC
AT2R	5'-AGTAAGCACAGAATTCAAAG	5'-AGTAAAGAATAGGAATTGCAT
GAPDH	5'-CTCTCTGCTCCTCCTGTTCGAC	5'-TGAGCGATGTGGCTCGGCT

### Determination of TGF-β, AngII or Aldo in Supernatant from Cultured HMC

TGF-β in supernatant from HMC culture was determined with the ELISA kits from R&D Systems (Minneapolis, MN, USA). The detection sensitivity was 32 pg/ml and the inter-batch coefficients of variation was 6.5%. AngII and Aldo were measured by an enzyme immunoassay kit (Cayman) according to the manufacturer's instruction. The minimum detectable concentration for AngII and Aldo was 1 and 7.8 pg/ml respectively with corresponding intra-assay coefficient of variation of 7 and 12.5%.

### Quantification of Apoptosis, Determination of ROS and NADPH Oxidase Activity

Activation of caspase 3 in PTEC cultured with IgA-HMC conditioned medium was determined using the caspase 3 activity fluorometric immunosorbant enzyme assay kit (Roche Diagnostics) according to manufacturer's protocol. ROS generation was measured with the fluoroprobe carboxymethyl-H_2_-dichlorofluorescein diacetate. Nicotinamide adenine dinucleotide phosphate (NADPH) oxidase activity was determined using an assay based on the chemiluminescence of lucigenin (bis-N-methylacridinium nitrate).

### Statistics

All data were expressed as means ± standard deviation (SD) unless otherwise specified. Statistical difference was analyzed with multivariate ANOVA for repeated measures. All p-values quoted are two-tailed and the significance is defined as p < 0.05.

## Results

### Expression of CYP11B2 and 11β-HSD2 in HMC and Their Regulation by pIgA

**Figure 1:**
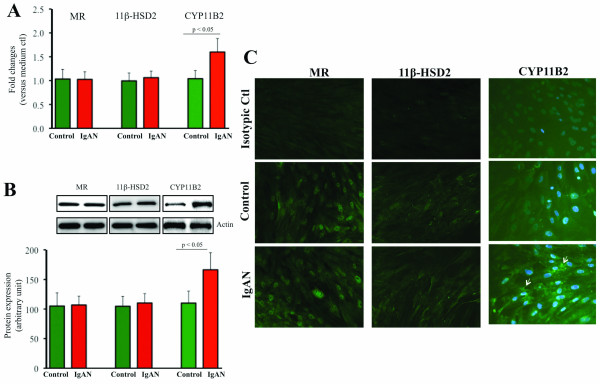
**Increased expression of CYP11B2 in HMC cultured with pIgA**. (A) There was increased expression of CYP11B2 mRNA and (B) CYP11B2 protein, but not 11β-HSD2, by HMC cultured with pIgA from patients (IgAN, n = 27) as compared to that of the controls (Control, n = 22). The results represent the mean ± SD. (C) Increased expression of CYP11B2 (arrow) in HMC was confirmed by immunofluorescence staining (magnification × 200).

**Figure 2:**
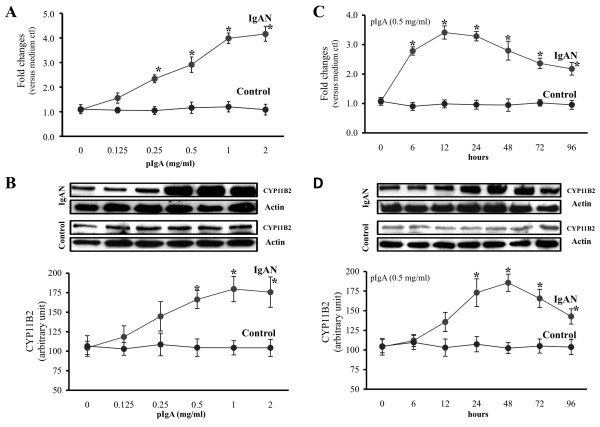
**Dose- and time-dependent CYP11B2 expression in HMC**. (A) Significant up-regulation of CYP11B2 mRNA expression and (B) CYP11B2 protein synthesis by HMC exposed to pIgA from IgAN patients (IgAN), but not with pIgA from controls (Control). (C) HMC cultured with pIgA (0.5 mg/ml) from IgAN, but not with pIgA from controls, exhibited an time-dependent increase of CYP11B2 mRNA expression (from 6 h, peaked at 12 h) and (D) CYP11B2 protein synthesis (from 24 h peaked at 48 h). The results represent the mean ± SD from five individual experiments. * signifies p < 0.05 when compared with data from HMC cultured in plain medium or data from time zero.

Cultured HMC expressed mRNA for the CYP11B2 and 11β-HSD2 constitutively and pIgA from IgAN patients up-regulated the gene (Figure 1A) and protein (Figure 1B) expression of CYP11B2 (p < 0.05), without altering the mRNA expression of 11β-HSD2. Up-regulation of CYP11B2 expression in HMC by pIgA from IgAN patients was confirmed by immunofluorescence staining (Figure 1C). A time- and dose-dependent increase in CYP11B2 expression in HMC was only demonstrated with pIgA from IgAN patients, but not with control pIgA (Figure 2).

### Aldo Up-regulated Mesangial Release of AngII

**Figure 3:**
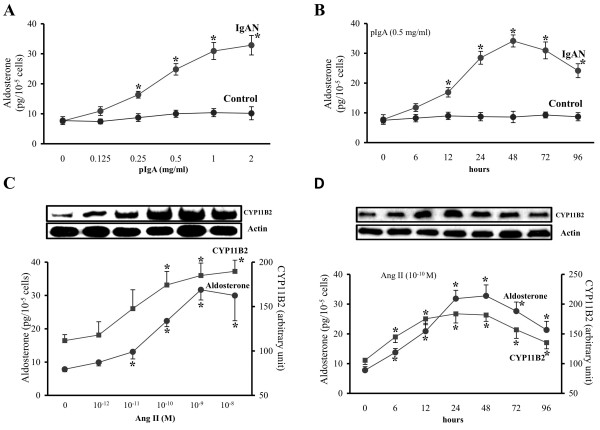
**Dose- and time-dependent Aldo release and CYP11B2 expression in HMC**. (A) Significant increase in Aldo release by HMC exposed to increasing concentration of pIgA from IgAN patients. (B) HMC cultured with pIgA (0.5 mg/ml) from IgAN patients, exhibited time-related increase in Aldo release (from 12 h, peaked at 48 h). (C) AngII induced Aldo release (at concentration > 10^-12 ^M) and CYP11B2 protein synthesis (at concentration > 10^-11 ^M) from HMC. (D) AngII (10^-10 ^M) induced time-related increase of Aldo release and CYP11B2 protein synthesis in HMC. The results represent the mean ± SD from five individual experiments. * signifies p < 0.05 when compared with data from HMC cultured in plain medium or data from time zero.

**Figure 4:**
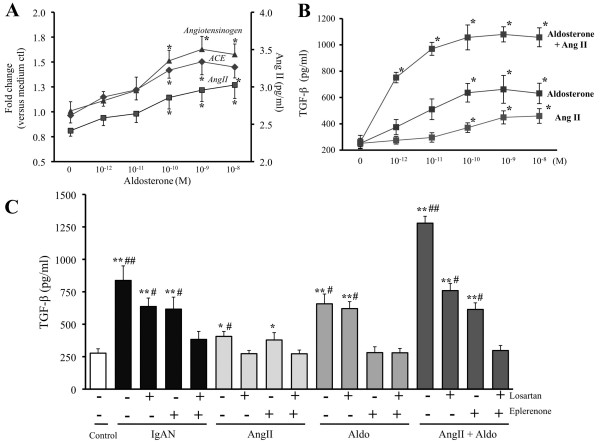
**Effect of Aldo or pIgA on TGF-β synthesis and RAS of HMC**. (A) Aldo at concentration >10^-11 ^M increased the mRNA expression of angiotensinogen and ACE, and AngII release by HMC. (B) AngII or Aldo at concentration >10^-11 ^M increased TGF-β synthesis by HMC. AngII and Aldo showed synergistic effect on TGF-β synthesis by HMC. * signifies p < 0.05 when compared with data from control without Aldo or AngII. (C) Pre-incubation with losartan (100 mM) or eplerenone (10 μM) one hour before cultured with pIgA from IgAN patients partially reduced the pIgA-induced TGF-β synthesis by HMC. Combining losartan and eplerenone completely abolished the increased TGF-β synthesis by pIgA. The AngII effect on TGF-β synthesis was blocked by losartan and the Aldo effect blocked by eplerenone; with complete normalization of TGF-β synthesis with the presence of both blockers. ** and * signify p < 0.01 and p < 0.05 respectively when compared with data from HMC cultured with pIgA from controls (Control). ## and # signify p < 0.01 and p < 0.05 respectively when compared with data from HMC pre-incubated with both inhibitors. The results represent the mean ± SD from five individual experiments.

Polymeric IgA from IgAN patients but not with pIgA from controls, at concentration > 0.25 mg/ml significantly increased the release of Aldo by HMC in a dose dependent manner (Figure 3A). At a concentration of 0.5 mg/ml, only pIgA from IgAN patients induced a time-dependent increase of Aldo in cultured HMC (Figure 3B). Exogenous AngII increased the Aldo release and CYP11B2 protein expression in a dose- and time-dependent manner (Figures 3C and 3D). Aldo at concentration > 10^-11 ^M increased the release of AngII and gene expression of angiotensinogen and ACE by HMC (Figure 4A).

### Role of Aldo and AngII in Regulation of TGF-β Synthesis by HMC

AngII and Aldo at concentration >10^-11 ^M individually increased TGF-β synthesis by HMC with synergistic effect (Figure 4B). Pre-incubation of HMC with either losartan or eplerenone partially reduced the pIgA-induced TGF-β synthesis by HMC (Figure 4C). The increased mesangial TGF-β synthesis induced by pIgA was completely abolished with dual blockade of losartan and eplerenone (Figure 4C).

### Expression of MR, but not CYP11B2 or 11β-HSD2 by PTEC

**Figure 5:**
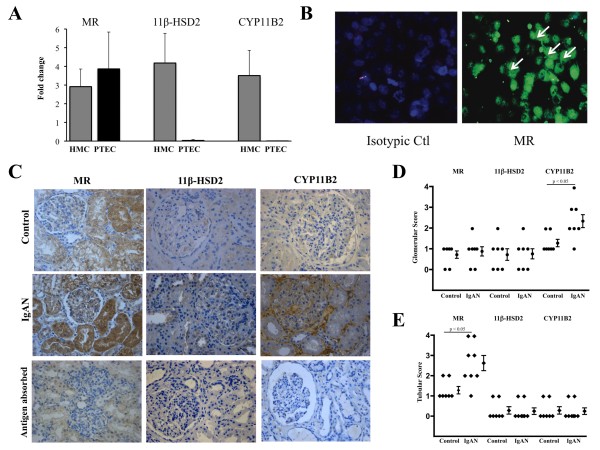
**Expression of MR, 11β-HSD2 or CYP11B2 in cultured HMC, PTEC and kidney biopsies**. (A) HMC expressed MR, 11β-HSD2 and CYP11B2 mRNAs and PTEC expressed only MR mRNA. Result was expressed as the mean fold change ± SD (related to the mean *C*_T _value from positive control using purified kidney total mRNA) from five individual experiments. (B) Demonstration of MR expression (arrows) in cultured PTEC by immunofluorescence staining (magnification × 200). (C). Detection of MR, 11β-HSD2 and CYP11B2 in kidney biopsies from IgAN patients and controls. Immunoreactive MR was located in both the glomeruli and tubules. Signal from immunoreactive 11β-HSD2 and CYP11B2 was located in glomeruli but not tubules (magnification × 200). Compared to staining results in control biopsies, increased (D) glomerular CYP11B2 staining and (E) tubular MR staining in IgAN patients were semi-quantified using a five-point scale.

Mineralocorticoid receptor mRNA was constitutively expressed in PTEC, but not for mRNA of CYP11B2 or 11β-HSD2 (Figure 5A). There was no induction of CYP11B2 or 11β-HSD2 gene expression in PTEC cultured with pIgA or conditioned medium from HMC incubated with pIgA from IgAN patients (IgA-HMC medium) (data not shown). Immunofluorescence staining confirmed the expression of MR in the resting PTEC (Figure 5B).

### Expression of MR, CYP11B2 or 11β-HSD2 in Human IgAN

Data on the expression of MR, CYP11B2 or 11β-HSD2 in cultured HMC or PTEC were further confirmed by immunohistochemical staining in kidney biopsies from patients with IgAN (Figure 5C). When compared with the staining results of the control biopsies, there was significantly increased expression of glomerular CYP11B2 and tubular MR in patients with IgAN (Figures 5D and 5E).

### Regulation of MR and AT2R expression in PTEC

**Figure 6:**
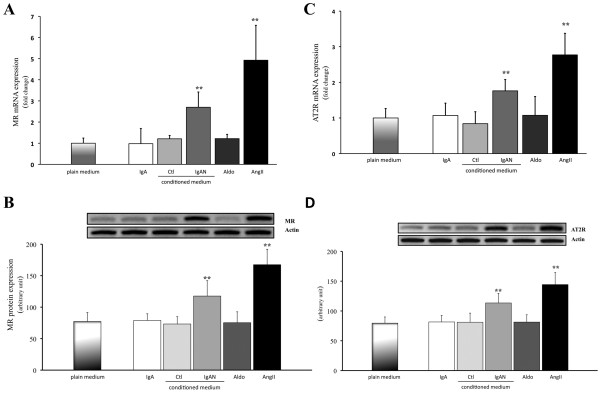
**Effect of pIgA, IgA-HMC conditioned medium, AngII and Aldo on the expression of MR or AT2R**. Growth arrested PTEC was cultured with (i) pIgA (IgA, 0.5 mg/ml) from IgAN patients; (ii) 4 fold diluted IgA-HMC medium prepared from controls or IgAN patients; (iii) Aldo (10^-10 ^M) or (iv) AngII (10^-10 ^M). Both the exogenous AngII and IgA-HMC medium prepared from IgAN patients significantly increased the expression of (A) MR mRNA, (B) MR protein, (C) AT2R mRNA and (D) AT2R protein as compared with PTEC cultured with plain medium control (** signifies p < 0.01). The results represent the mean ± SD from five individual experiments.

**Figure 7:**
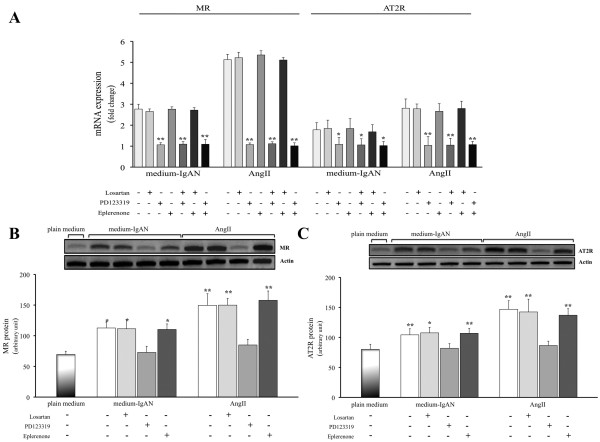
**The effect of MR, AT1R or AT2R blockade on IgA-HMC medium or AngII induced MR or AT2R expression**. PD123319 effectively abolished the up-regulation of MR and AT2R receptor mRNA (A) or protein expression (B & C) by IgA-HMC medium from IgAN patients (medium-IgA) or AngII. Neither losartan nor eplerenone altered these receptors expression in PTEC. * and ** signify p < 0.05 and p < 0.01 respectively when compared with data from PTEC cultured in plain medium. The results represent the mean ± SD from five individual experiments.

Both exogenous AngII and IgA-HMC medium prepared from IgAN patients significantly increased the expression of MR and AT2R in PTEC (Figures 6A to 6D) as compared with plain medium. The AT2R antagonist, PD123319, effectively abolished the enhanced expression of MR (Figure 7). In contrast, neither losartan nor eplerenone exhibited any inhibitory action.

### AngII, Aldo or IgA-HMC Medium from IgAN Patients Induced Apoptosis in PTEC

**Figure 8:**
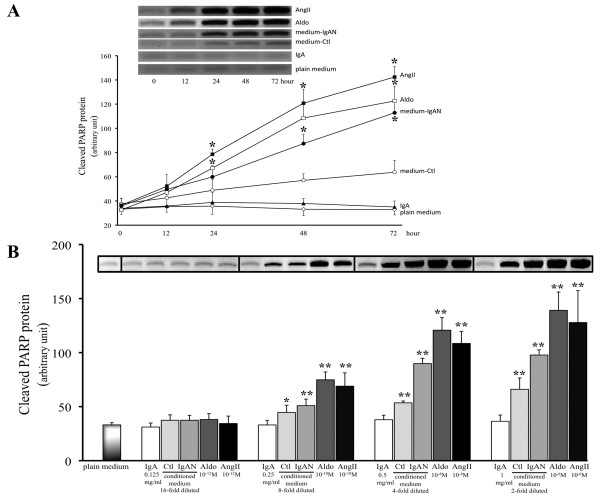
**Time- and dose-related expression of cleaved PARP by PTEC**. (A) Up-regulation of cleaved PARP expression by PTEC cultured with Aldo (10^-10 ^M), AngII (10^-10 ^M) or 4 fold diluted IgA-HMC medium prepared from IgAN patients (medium-IgAN). Similar up-regulation was not observed with IgA-HMC medium from controls (medium-Ctl) or pIgA alone (IgA). (B) Dose-dependent up-regulation of cleaved PARP expression by PTEC cultured with Aldo, AngII or IgA-HMC medium prepared from IgAN patients. * and ** signify p < 0.05 and p < 0.01 respectively when compared with data from time zero or from PTEC cultured in plain medium. The results represent the mean ± SD from five individual experiments.

**Figure 9:**
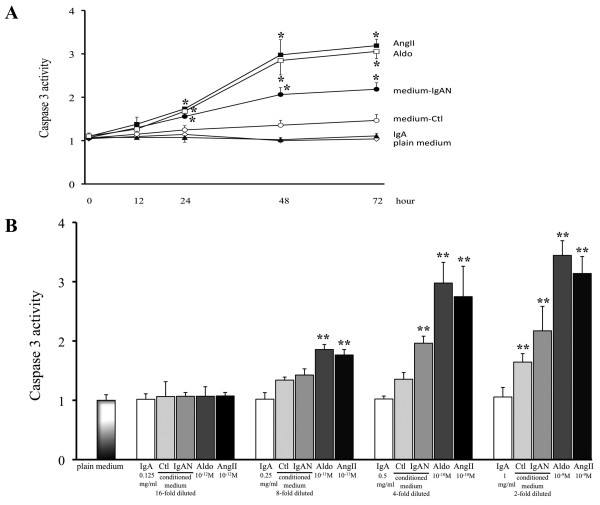
**Time- and dose-related assay of caspase 3 activity in PTEC**. (A) Up-regulation of caspase 3 activity by PTEC cultured with Aldo (10^-10 ^M), AngII (10^-10 ^M) or 4 fold diluted IgA-HMC medium prepared from IgAN patients (medium-IgAN); Similar up-regulation was not observed with IgA-HMC medium from controls (medium-Ctl) or pIgA alone (IgA). (B) Dose-dependent up-regulation of caspase 3 activity by PTEC cultured with Aldo, AngII or IgA-HMC medium prepared from IgAN patients. * and ** signify p < 0.05 and p < 0.01 respectively when compared with data from time zero or from PTEC cultured in plain medium. The results represent the mean ± SD from five individual experiments.

**Figure 10:**
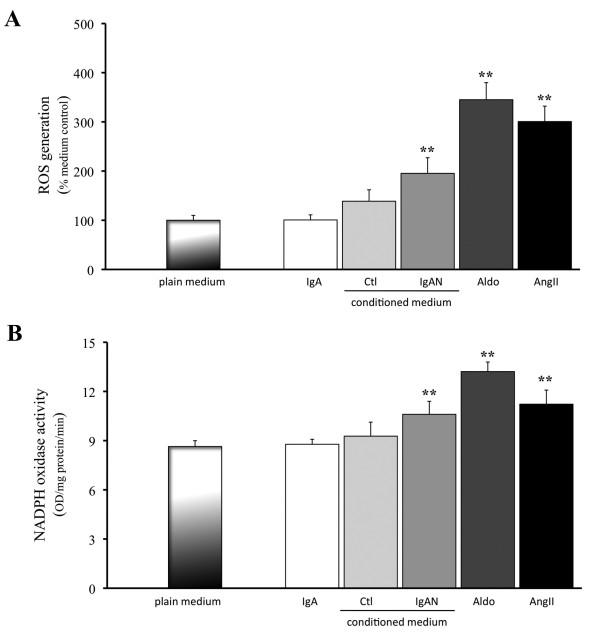
**ROS generation and NADPH oxidase activity by PTEC**. (A) Up-regulation of ROS generation and (B) NADPH oxidase activity by PTEC cultured with Aldo (10^-10 ^M), AngII (10^-10 ^M) or 4 fold diluted IgA-HMC medium prepared from IgAN patients (IgAN); Similar up-regulation was not observed with IgA-HMC medium from controls (Ctl) or pIgA alone (IgA). ** signify p < 0.01 when compared with data from PTEC cultured in plain medium. The results represent the mean ± SD from five individual experiments.

**Figure 11:**
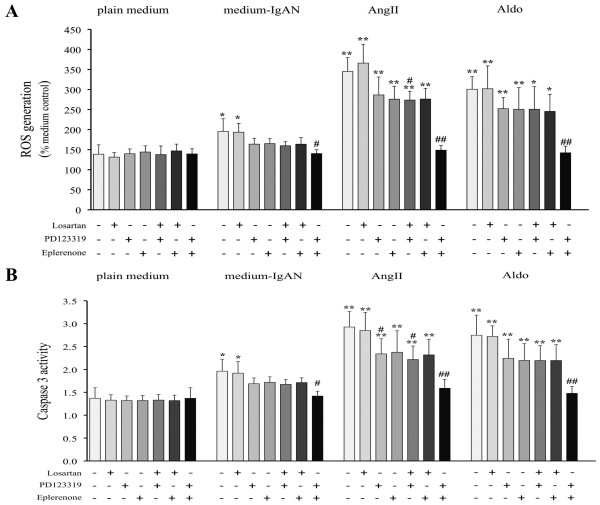
**The effect of MR, AT1R or AT2R blockade in ROS generation and caspase 3 activity by PTEC**. (A) Individual use of MR, AT1R or AT2R antagonist failed to effectively inhibit the up-regulation of ROS generation and (B) caspase 3 activity by PTEC cultured with Aldo (10^-10 ^M), AngII (10^-10 ^M) or 4 fold diluted IgA-HMC medium prepared from IgAN patients (medium-IgAN). Abolishment of up-regulated ROS generation and caspase 3 activity by PTEC was only achieved by combining PD123319 and eplerenone. * and ** signify p < 0.05 and p < 0.01 respectively when compared with data from HMC cultured with plain medium control. ## and # signify p < 0.01 and p < 0.05 respectively when compared with data from HMC corresponding activator without pre-incubation of any receptor antagonist. The results represent the mean ± SD from five individual experiments.

PTEC cultured with Aldo (10^-10 ^M), AngII (10^-10 ^M) or 4-fold diluted IgA-HMC medium prepared from IgAN patients, induced cellular apoptosis as indicated by time-and dose-related up-regulation of cleaved PARP expression (Figures 8A and 8B) and caspase 3 activity (Figures 9A and 9B). Similar findings were not observed with conditioned medium prepared from controls or direct culture with pIgA from IgAN patients. The induced apoptosis in PTEC was associated with increased reactive oxygen species (ROS) generation and NAPDH oxidase activity (Figures 10A and 10B). The ROS generation and caspase 3 activity induced by Aldo, AngII or IgA-HMC medium prepared from IgAN patients were partially suppressed by PD123319 or eplerenone and completely abolished with combined use of PD123319 and eplerenone (Figure 11). AT1R blockade with losartan exhibited no suppressive effect.

## Discussion

In this study, *in vitro *culture models of HMC and PTEC were used to examine Aldo synthesis by pIgA directly or through glomerular-tubular communications. The possible role of Aldo on PTEC injury and its interaction with AngII in IgAN was also elucidated. The RAS has been implicated in the development of progressive glomerulosclerosis in diabetic and non-diabetic nephropathy [22,23]. We had previously demonstrated an increased expression of renin and TGF-β by HMC incubated with pIgA from IgAN patients [8]. Polymeric IgA from these patients also up-regulated the Smad activity in HMC which was readily suppressed with captopril or losartan, supporting a pathogenetic role of pIgA1 in IgAN through up-regulation of the RAS and TGF-β. Growing evidence from experimental and clinical studies indicates that increased Aldo is an independent contributor to arterial injury and nephropathy [24]. Both human and rat mesangial cells produce Aldo in culture [25,26]. Exogenous Aldo significantly increased fibronectin production by mesangial cells [26]. Renal TGF-ß and fibronectin are increased in Dahl salt-sensitive rat with heart failure (DSHF rat) in association with the development of glomerulosclerosis [27]. Aldo blocker significantly suppressed renal TGF-ß expression in DSHF rat with reduced glomerulosclerosis. *In vivo *study also showed Aldo administration *per se *induced TGF-ß release and organ fibrosis independent of blood pressure [28]. In the present study, we demonstrate that Aldo may initiate and amplify injury to HMC upon activation by pIgA in both paracrine and endocrine manner. We also reveal a vicious cycle between pIgA-induced AngII and Aldo amplifying the TGF-β synthesis *via *binding to AT1R and MR in cultured HMC in the setting mimicking IgAN.

Using similar *in vitro *culture model, we had previously demonstrated that that tumor necrosis factor-α (TNF-α) and AngII are released from mesangial cells following initial deposition of "pathogenetic" pIgA [3]. This is followed by mesangial proliferation and glomerular infiltration of immuno-competent cells. A cross-talk network initiated by TNF-α, AngII and other soluble factors orchestrates interactions between infiltrating immuno-competent cells and the resident renal cells, forming a major driving force of tubulointerstitial injury. We further demonstrate that AngII, but not Aldo, released by pIgA-activated HMC up-regulate the expression of AT2R and MR by PTEC. The increased MR expression in PTEC is readily available for the binding to Aldo, released by the pIgA-activated HMC, to initiate the downstream events of PTEC injury. Our observation of an up-regulation of MR but not 11β-HSD2 in PTEC is intriguing. Previously, it is believed that the specificity for MR to Aldo is conferred enzymatically by the cortisol-inactivating enzyme 11β-HSD2 [29,30]. However, recent evidence has suggested that it may not be totally true as it is improbable for the intrarenal 11β-HSD2 to convert all the glucocorticoid molecules to MR-inactive metabolites with the inexhaustible supply of steroid substrate [31]. The current view suggests that the enzyme 11β-HSD2 does not block the glucocorticoid occupancy of MR but the ability of glucocorticoid to act as MR agonist. In the absence of the enzyme, there is intracellular redox change due to reduced generation of nicotinamide-adenine dinucleotide (NADH) from nicotinamide-adenine dinucleotide (NAD), the co-substrate for the cortisol-to-cortisone conversion [31,32]. It has been demonstrated by patch-clamp study in isolated rabbit cardiomyocytes that under normal conditions, cortisol is an MR antagonist on the Aldo-induced transmembrane pump current. However, cortisol becomes an MR agonist, mimicking the effect of aldosterone, under redox change with instillation of oxidized glutathione [33]. It has been suggested that MR, which are constitutively occupied by normal glucocorticoid levels, responds to changes in intracellular redox state in tissues either expressing (e.g. vascular smooth muscle cells) [34] or lacking 11β-HSD2 (e.g. cardiomyocytes) [35]. We speculate that apart from the increased availability of MR expression to the up-regulated aldosterone release by pIgA-activated HMC in PTEC, cellular cortisol in PTEC may also act agonistically on the up-regulated MR in the absence of 11β-HSD2 under redox changes. The combined effect of aldosterone and cortisol *via *MR may eventually lead to cellular apoptosis.

Our data reveal that PTEC cultured with AngII, Aldo or IgA-HMC medium prepared from IgAN patients express increased NADPH oxidase and ROS formation. The events are associated with increased apoptosis in PTEC demonstrated by dose- and time-dependent up-regulation of cleaved PARP expression and caspase 3 activity. The primary function NADPH oxidase is to generate ROS [36,37]. Although the NADPH oxidase was originally identified in phagocytic leukocytes, recent study discovered that this enzyme is an essential endogenous origin of cellular oxidative stress and cellular injury in other cell types [36]. Furthermore, ROS generated by the NADPH oxidase may also modulate many intracellular redox signal transduction pathways [36]. PTEC process all components of the NADPH oxidase complex [36,38,39]. The exact function of NADPH oxidase in regulating physiological and pathophysiological processes in the kidney remains unknown. Data from *in vitro *experiments have shown that NADPH oxidase-derived ROS mediate apoptosis in renal cells [39,40]. AngII is an important *in vivo *regulator of NADPH oxidase in the kidney through activation of renal NADPH oxidase and induction of oxidative stress [38]. Recent data from transgenic mouse model suggest that up-regulation of NADPH oxidase-dependent ROS generation is important in mediating apoptosis in renal tubular epithelial cell induced by AngII. Apart from AngII, our present study provides novel *in vitro *data showing that Aldo also induces NADPH oxidase expression and its dependent ROS generation by PTEC. Further *in vivo *experiment is needed to validate the role of Aldo in inducing PTEC apoptosis through NADPH oxidase and ROS generation.

Blockade of the RAS with ACEI or AT1R blocker protects hypertensive and diabetic kidney injuries by inhibiting the renal NADPH [41-43]. Aldo also enhances NADPH oxidase activity via translocation of the cytosolic component, p47phox, to the membrane subunits by an ERK1/2-related pathway [44]. Aldo has also been shown to stimulate angiotensin-converting enzyme (ACE) expression which stimulates renal AngII production [45]. Recent clinical studies indicate that Aldo blockade may provide renoprotection by decreasing proteinuria, independent of blood pressure control. The combination of ACEI and the selective Aldo blocker, eplerenone, can provide a better renal outcome with reduction of proteinuria and kidney damage than monotherapy [24,46]. Interestingly, treatment with eplerenone alone or combining with the ACEI reduced proteinuria more effectively than the ACEI alone in human studies [47,48]. In DSHF rat, combined therapy with eplerenone and ACEI reduced glomerulosclerosis, proteinuria and improved renal function more effectively than monotherapy [27]. Based on the finding of up-regulated expression of MR and AT2R in PTEC by IgA-HMC medium from IgAN patients and AngII, we tested whether combined blockade of AT2R and MR could suppress pIgA-induced PTEC apoptosis through the glomerulo-tubular communication. Our present data confirm that blockade with PD123319 and eplererone abolishes NADPH-dependent ROS generation and apoptosis. Further *in vivo *study is needed to test whether combined AngII receptor and Aldo blockade provides a better renal outcome with reduction of proteinuria and tissue damage in IgAN.

**Figure 12:**
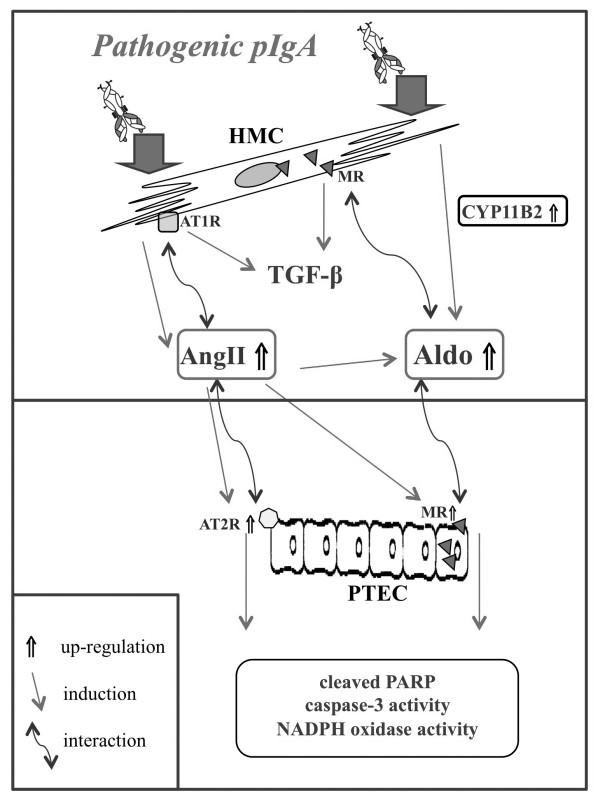
**Schema showing the possible role of RAAS components in the glomerulo-tubular communication operating the pathogenesis of IgAN**. Polymeric IgA from IgAN patients is capable of enhancing AngII release or Aldo synthesis through increasing the expression of ACE/ANG or CYP11B2 in HMC. The increased synthesis of AngII and Aldo up-regulates the TGF-β synthesis *via *binding to their respective receptors: AT1R and MR. Aldo is able to further up-regulate the ACE/ANG expression and AngII release in HMC whereas AngII increases the Aldo release by HMC *via *increased expression of CYP11B2. This vicious cycle involving pIgA-induced AngII and Aldo in HMC amplifies the pIgA-induced HMC damages and initiates the downstream inflammatory cascade in PTEC through the glomerulo-tubular communication. AngII released by pIgA-activated HMC up-regulates the expression of AT2R and MR by PTEC. Binding of AngII and Aldo to their respective receptors on PTEC induces cellular apoptosis through increased NADPH activity and generation of ROS. Combined blockade of AT2R and MR can prevent pIgA-induced PTEC apoptosis through these glomerulo-tubular communications.

Based on our previous studies [3,4,8,9,49] and the present data, a mechanistic schema summarizing the contributory role of the RAAS in glomerulo-tubular communications operating in tubulointerstitial injury in IgAN is shown in Figure 12.

## Conclusions

Taken together, our *in vitro *data suggest that AngII and Aldo, released by pIgA1 activated HMC, are mediators for glomerulotubular crosstalk in inducing apoptosis of PTEC through NADPH dependent generation of ROS. In addition, crosstalk between AngII and Aldo could play a role in pIgA1 induced PTEC apoptosis in IgAN.

## Competing interests

The authors declare that they have no competing interests.

## Authors' contributions

All authors have read and approved the final manuscript. JCKL and KNL designed the experiment, drafted the experiments and troubleshooting. JCKL, LYYC, CWC, and AIL were responsible for the laboratory assay and performing the experiments. JCKL, MFL, SCWT and KNL participated in refinement of experiment protocol and coordination and helped in drafting the manuscript.

## References

[B1] D'Amico G The commonest glomerulonephritis in the world: IgA nephropathy Q J Med 1987 64 709 727 3329736

[B2] D'Amico G Ferrario F Rastaldi MP Tubulointerstitial damage in glomerular diseases: its role in the progression of renal damage Am J Kidney Dis 1995 26 124 132 10.1016/0272-6386(95)90165-5 7611242

[B3] Chan LY Leung JC Tsang AW Tang SC Lai KN Activation of tubular epithelial cells by mesangial-derived TNF-alpha: glomerulotubular communication in IgA nephropathy Kidney Int 2005 67 602 612 10.1111/j.1523-1755.2005.67116.x 15673307

[B4] Chan LY Leung JC Tang SC Choy CB Lai KN Tubular expression of angiotensin II receptors and their regulation in IgA nephropathy J Am Soc Nephrol 2005 16 2306 2317 10.1681/ASN.2004121117 15930094

[B5] Coppo R Amore A Gianoglio B Cacace G Picciotto G Roccatello D Peruzzi L Piccoli G De Filippi PG Angiotensin II local hyperreactivity in the progression of IgA nephropathy Am J Kidney Dis 1993 21 593 602 8503412 10.1016/s0272-6386(12)80031-x

[B6] Del Prete D Gambaro G Lupo A Anglani F Brezzi B Magistroni R Graziotto R Furci L Modena F Bernich P Precocious activation of genes of the renin-angiotensin system and the fibrogenic cascade in IgA glomerulonephritis Kidney Int 2003 64 149 159 10.1046/j.1523-1755.2003.00065.x 12787405

[B7] Remuzzi A Fassi A Bertani T Perico N Remuzzi G ACE inhibition induces regression of proteinuria and halts progression of renal damage in a genetic model of progressive nephropathy Am J Kidney Dis 1999 34 626 632 10.1016/S0272-6386(99)70385-9 10516341

[B8] Lai KN Tang SC Guh JY Chuang TD Lam MF Chan LY Tsang AW Leung JC Polymeric IgA1 from patients with IgA nephropathy upregulates transforming growth factor-beta synthesis and signal transduction in human mesangial cells via the renin-angiotensin system J Am Soc Nephrol 2003 14 3127 3137 10.1097/01.ASN.0000095639.56212.BF 14638911

[B9] Lai KN Chan LY Tang SC Tsang AW Li FF Lam MF Lui SL Leung JC Mesangial expression of angiotensin II receptor in IgA nephropathy and its regulation by polymeric IgA1 Kidney Int 2004 66 1403 1416 10.1111/j.1523-1755.2004.00874.x 15458433

[B10] Nishiyama A Abe Y Aldosterone and renal injury Nippon Yakurigaku Zasshi 2004 124 101 109 10.1254/fpj.124.101 15277728

[B11] Mackenzie SM Connell J Hypertension and the expanding role of aldosterone Curr Hypertens Rep 2006 8 255 261 10.1007/s11906-006-0059-y 17147925

[B12] Hostetter TH Rosenberg ME Ibrahim HN Juknevicius I Aldosterone in progressive renal disease Semin Nephrol 2001 21 573 579 10.1053/snep.2001.26797 11709805

[B13] Connell JM Davies E The new biology of aldosterone J Endocrinol 2005 186 1 20 10.1677/joe.1.06017 16002531

[B14] Greene EL Kren S Hostetter TH Role of aldosterone in the remnant kidney model in the rat J Clin Invest 1996 98 1063 1068 10.1172/JCI118867 8770880 PMC507523

[B15] Blasi ER Rocha R Rudolph AE Blomme EA Polly ML McMahon EG Aldosterone/salt induces renal inflammation and fibrosis in hypertensive rats Kidney Int 2003 63 1791 1800 10.1046/j.1523-1755.2003.00929.x 12675855

[B16] Rocha R Rudolph AE Frierdich GE Nachowiak DA Kekec BK Blomme EA McMahon EG Delyani JA Aldosterone induces a vascular inflammatory phenotype in the rat heart Am J Physiol Heart Circ Physiol 2002 283 H1802 1810 12384457 10.1152/ajpheart.01096.2001

[B17] Luther JM Gainer JV Murphey LJ Yu C Vaughan DE Morrow JD Brown NJ Angiotensin II induces interleukin-6 in humans through a mineralocorticoid receptor-dependent mechanism Hypertension 2006 48 1050 1057 10.1161/01.HYP.0000248135.97380.76 17043157

[B18] Bianchi S Bigazzi R Campese VM Antagonists of aldosterone and proteinuria in patients with CKD: an uncontrolled pilot study Am J Kidney Dis 2005 46 45 51 10.1053/j.ajkd.2005.03.007 15983956

[B19] To WY Leung JC Lai KN Identification and characterization of human serum alpha2-HS glycoprotein as a jacalin-bound protein Biochim Biophys Acta 1995 1249 58 64 10.1016/0167-4838(95)00063-Z 7766684

[B20] Lai KN To WY Li PK Leung JC Increased binding of polymeric lambda-IgA to cultured human mesangial cells in IgA nephropathy Kidney Int 1996 49 839 845 10.1038/ki.1996.116 8648928

[B21] Tam KY Leung JC Chan LY Lam MF Tang SC Lai KN In vitro enhanced chemotaxis of CD25+ mononuclear cells in patients with familial IgAN through glomerulotubular interactions Am J Physiol Renal Physiol 2010 299 F359 368 10.1152/ajprenal.00664.2009 20484297

[B22] Leehey DJ Singh AK Alavi N Singh R Role of angiotensin II in diabetic nephropathy Kidney Int Suppl 2000 77 S93 98 10997697 10.1046/j.1523-1755.2000.07715.x

[B23] Durvasula RV Shankland SJ The renin-angiotensin system in glomerular podocytes: mediator of glomerulosclerosis and link to hypertensive nephropathy Curr Hypertens Rep 2006 8 132 138 10.1007/s11906-006-0009-8 16672146

[B24] Hollenberg NK Aldosterone in the development and progression of renal injury Kidney Int 2004 66 1 9 10.1111/j.1523-1755.2004.00701.x 15200407

[B25] Nishikawa T Suematsu S Saito J Soyama A Ito H Kino T Chrousos G Human renal mesangial cells produce aldosterone in response to low-density lipoprotein (LDL) J Steroid Biochem Mol Biol 2005 96 309 316 10.1016/j.jsbmb.2005.03.005 15993578

[B26] Lai L Chen J Hao CM Lin S Gu Y Aldosterone promotes fibronectin production through a Smad2-dependent TGF-beta1 pathway in mesangial cells Biochem Biophys Res Commun 2006 348 70 75 10.1016/j.bbrc.2006.07.057 16876110

[B27] Onozato ML Tojo A Kobayashi N Goto A Matsuoka H Fujita T Dual blockade of aldosterone and angiotensin II additively suppresses TGF-beta and NADPH oxidase in the hypertensive kidney Nephrol Dial Transplant 2007 22 1314 1322 10.1093/ndt/gfl780 17324946

[B28] Juknevicius I Segal Y Kren S Lee R Hostetter TH Effect of aldosterone on renal transforming growth factor-beta Am J Physiol Renal Physiol 2004 286 F1059 1062 10.1152/ajprenal.00202.2003 15130897

[B29] Funder JW Pearce PT Smith R Smith AI Mineralocorticoid action: target tissue specificity is enzyme, not receptor, mediated Science 1988 242 583 585 10.1126/science.2845584 2845584

[B30] Edwards CR Stewart PM Burt D Brett L McIntyre MA Sutanto WS de Kloet ER Monder C Localisation of 11 beta-hydroxysteroid dehydrogenase--tissue specific protector of the mineralocorticoid receptor Lancet 1988 2 986 989 2902493 10.1016/s0140-6736(88)90742-8

[B31] Funder JW Reconsidering the roles of the mineralocorticoid receptor Hypertension 2009 53 286 290 19139379 10.1161/HYPERTENSIONAHA.108.119966

[B32] Fjeld CC Birdsong WT Goodman RH Differential binding of NAD+ and NADH allows the transcriptional corepressor carboxyl-terminal binding protein to serve as a metabolic sensor Proc Natl Acad Sci USA 2003 100 9202 9207 10.1073/pnas.1633591100 12872005 PMC170896

[B33] Funder JW RALES, EPHESUS and redox J Steroid Biochem Mol Biol 2005 93 121 125 10.1016/j.jsbmb.2004.12.010 15860254

[B34] Alzamora R Michea L Marusic ET Role of 11beta-hydroxysteroid dehydrogenase in nongenomic aldosterone effects in human arteries Hypertension 2000 35 1099 1104 10818071 10.1161/01.hyp.35.5.1099

[B35] Mihailidou AS Loan Le TY Mardini M Funder JW Glucocorticoids activate cardiac mineralocorticoid receptors during experimental myocardial infarction Hypertension 2009 54 1306 1312 10.1161/HYPERTENSIONAHA.109.136242 19841288

[B36] Bedard K Krause KH The NOX family of ROS-generating NADPH oxidases: physiology and pathophysiology Physiol Rev 2007 87 245 313 10.1152/physrev.00044.2005 17237347

[B37] Jiang F NADPH oxidase in the kidney: a Janus in determining cell fate Kidney Int 2009 75 135 137 10.1038/ki.2008.478 19116641

[B38] Gill PS Wilcox CS NADPH oxidases in the kidney Antioxid Redox Signal 2006 8 1597 1607 10.1089/ars.2006.8.1597 16987014

[B39] Lodha S Dani D Mehta R Bhaskaran M Reddy K Ding G Singhal PC Angiotensin II-induced mesangial cell apoptosis: role of oxidative stress Mol Med 2002 8 830 840 12606818 PMC2039960

[B40] Susztak K Raff AC Schiffer M Bottinger EP Glucose-induced reactive oxygen species cause apoptosis of podocytes and podocyte depletion at the onset of diabetic nephropathy Diabetes 2006 55 225 233 10.2337/diabetes.55.01.06.db05-0894 16380497

[B41] Tojo A Asaba K Onozato ML Suppressing renal NADPH oxidase to treat diabetic nephropathy Expert Opin Ther Targets 2007 11 1011 1018 10.1517/14728222.11.8.1011 17665974

[B42] Onozato ML Tojo A Goto A Fujita T Wilcox CS Oxidative stress and nitric oxide synthase in rat diabetic nephropathy: effects of ACEI and ARB Kidney Int 2002 61 186 194 10.1046/j.1523-1755.2002.00123.x 11786100

[B43] Hanna IR Taniyama Y Szocs K Rocic P Griendling KK NAD(P)H oxidase-derived reactive oxygen species as mediators of angiotensin II signaling Antioxid Redox Signal 2002 4 899 914 10.1089/152308602762197443 12573139

[B44] Nishiyama A Yao L Nagai Y Miyata K Yoshizumi M Kagami S Kondo S Kiyomoto H Shokoji T Kimura S Possible contributions of reactive oxygen species and mitogen-activated protein kinase to renal injury in aldosterone/salt-induced hypertensive rats Hypertension 2004 43 841 848 10.1161/01.HYP.0000118519.66430.22 14769808

[B45] Harada E Yoshimura M Yasue H Nakagawa O Nakagawa M Harada M Mizuno Y Nakayama M Shimasaki Y Ito T Aldosterone induces angiotensin-converting-enzyme gene expression in cultured neonatal rat cardiocytes Circulation 2001 104 137 139 11447075 10.1161/01.cir.104.2.137

[B46] Sato A Saruta T Funder JW Combination therapy with aldosterone blockade and renin-angiotensin inhibitors confers organ protection Hypertens Res 2006 29 211 216 10.1291/hypres.29.211 16778327

[B47] Brennan BJ Martin NE Eplerenone: selective aldosterone antagonism in management of cardiovascular and renal disease J Am Pharm Assoc (2003) 2004 44 604 610 10.1331/1544-3191.44.5.604.Brennan 15496047

[B48] Williams GH Burgess E Kolloch RE Ruilope LM Niegowska J Kipnes MS Roniker B Patrick JL Krause SL Efficacy of eplerenone versus enalapril as monotherapy in systemic hypertension Am J Cardiol 2004 93 990 996 10.1016/j.amjcard.2004.01.007 15081441

[B49] Lai KN Chan LY Leung JC Mechanisms of tubulointerstitial injury in IgA nephropathy Kidney Int Suppl 2005 S110 115 10.1111/j.1523-1755.2005.09426.x 15752226

